# Continuous Glucose Monitoring in Glycogen Storage Diseases: A Systematic Review of Clinical Utility, Accuracy and Patient Outcomes

**DOI:** 10.1002/edm2.70274

**Published:** 2026-07-02

**Authors:** Reihaneh Mohsenipour, Farzaneh Abbasi, Maryam Sadat Ghaderian, Asal Khalili Dehkordi, Saeideh Abdolahpour, Kasra Akbari

**Affiliations:** ^1^ Growth and Development Research Center, Children’s Medical Center Tehran University of Medical Sciences Tehran Iran; ^2^ Children’s Medical Center Tehran University of Medical Sciences Tehran Iran

**Keywords:** continuous glucose monitoring, glycogen storage disease, hypoglycemia, metabolic control

## Abstract

**Background:**

Achieving euglycemia in Glycogen Storage Diseases (GSD) depends on strict dietary adherence; however, intermittent monitoring frequently fails to detect rapid hypoglycemic excursions. This systematic review evaluates the clinical utility, accuracy and psychosocial impact of Continuous Glucose Monitoring (CGM) across hepatic GSD subtypes.

**Methods:**

We searched PubMed, Embase, Scopus, Web of Science and Cochrane Library (inception to December 2025) following PRISMA 2020 guidelines. Eligible studies included randomized trials, cohorts and case series reporting CGM outcomes in hepatic GSD.

**Results:**

Thirty‐one studies (*N* = 323) met inclusion criteria, predominantly GSD Ia, Ib and III. CGM consistently identified asymptomatic hypoglycemia, with adult cohorts spending up to 12% of time below range (< 70 mg/dL) despite normal spot checks. In interventional settings, CGM‐guided dietary titration improved Time in Range (e.g., 72%–86% in gene therapy trials) and reduced serum triglycerides and liver size. However, sensor performance showed a positive bias in the hypoglycemic range, potentially masking severe neuroglycopenia. Psychosocially, while CGM improved sleep quality scores, it introduced ‘alarm fatigue’ from false‐positive alerts.

**Conclusion:**

Emerging evidence suggests that CGM may shift GSD management from reactive treatment to proactive stabilization. It offers superior sensitivity for detecting occult hypoglycemia and guiding therapy, though its utility is tempered by sensor inaccuracy at lower limits and the burden of constant surveillance. Future implementation requires subtype‐specific interpretation and sensors optimized for the hypoglycemic range.

## Graphical abstract

This review shows that CGM can shift GSD management from reactive surveillance to proactive metabolic regulation, significantly improving Time in Range. While effective in detecting occult hypoglycemia, its clinical utility is currently limited by low‐range sensor inaccuracies and alarm fatigue, necessitating individualized patient management.

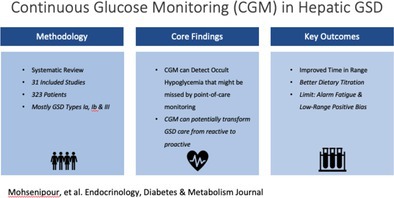

## 1 Introduction

The clinical management of Glycogen Storage Diseases (GSD) requires strict euglycemia to avert the activation of counter‐regulatory pathways [1, 2]. In the practice guidelines by Kishnani et al. [3], the main therapeutic goal is to keep blood glucose levels above 70 mg/dL to prevent acute neuroglycopenia and suppress secondary biochemical surges of lactate, uric acid and lipids that cause long‐term morbidity. Wang et al. [4] demonstrated that chronic metabolic instability is a key driver of hepatocellular adenoma formation, while Melis et al. [5] linked poor glycemic control to the progression of renal injury and growth failure. As a result, the cornerstone of GSD treatment remains a rigorous dietary schedule of frequent complex carbohydrate administration. To avoid hypoglycemia, a constant, accurate monitoring plan is essential.

However, the feasibility of maintaining such rigorous glucose targets is frequently compromised by the inherent limitations of intermittent monitoring. Traditional self‐monitoring of blood glucose (SMBG) provides only static snapshots of metabolic control, blinding clinicians to the dynamic fluctuations that occur during the critical overnight fasting period and between meals [6]. Many individuals termed ‘stable’ by conventional fingerstick testing really have asymptomatic hypoglycemia, with studies showing that 4%–8% of daily time is spent below the safety threshold of 70 mg/dL without detection [7]. Counter‐regulatory mechanisms can mask the adrenergic symptoms of hypoglycemia, causing neuroglycopenia to last up to 60% of monitoring time in some adult cohorts while they remain asymptomatic and falsely reassured by normal spot checks [8].

Thanks to Continuous Glucose Monitoring (CGM) systems, GSD care can be shifted from reactive treatment of symptoms to proactive glucose stabilization [9, 10, 11, 12]. This continuous data stream allows clinicians to visualize the immediate impact of dietary interventions, fine‐tune nocturnal enteral feeding rates and detect early trends toward hypoglycemia before they become critical [13, 14, 15]. Furthermore, by shifting monitoring predominantly to sensor‐based readings, CGM reduces the physical and psychological toll of frequent SMBG. Evidence from various clinical settings demonstrates that this transition can reduce the need for bedside capillary fingerstick checks and associated nursing time by roughly 40%–60%, maintaining safety while substantially lowering the burden of painful procedures [16, 17, 18]. However, GSD management operates almost exclusively at the lower limit of sensor detection, where signal noise, lag time and ‘alarm fatigue’ from false‐positive alerts present challenges to clinical interpretation and caregiver burden [6, 19].

CGM's usage in GSDs is growing; however, the literature is still fragmented, with much of the data coming from case reports. Recent studies have addressed CGM throughout the range of inherited metabolic diseases [20], but a comprehensive, up‐to‐date synthesis on GSD's particular glycemic and psychosocial needs is needed. This systematic review examines CGM in the hypoglycemia range and its increasing relevance as a key endpoint in gene therapy studies to fill that gap. This study integrates accuracy, metabolic outcomes, patient‐reported quality of life and device restrictions to give physicians a clearer, evidence‐based framework for adopting continuous monitoring as a standard of care to optimize long‐term health outcomes.

## 2 Methods

### 2.1 Protocol and Registration

This systematic review was conducted in accordance with the Preferred Reporting Items for Systematic Reviews and Meta‐Analyses (PRISMA) 2020 guidelines. The protocol was designed to evaluate the clinical utility, accuracy and psychosocial impact of CGM and Flash Glucose Monitoring (FGM) in patients with GSD. However, the review protocol was not prospectively registered in a registry such as PROSPERO due to the exploratory scope of the initial literature search, which aimed to accommodate the high heterogeneity of available evidence.

### 2.2 Search Strategy

A comprehensive systematic search was performed across five major electronic databases: PubMed, Embase, Scopus, Web of Science and the Cochrane Library, covering literature from inception to December 14, 2025. The search strategy utilized a combination of controlled vocabulary (MeSH/Emtree) and free‐text keywords focused on two primary concepts: (1) Population (e.g., ‘Glycogen Storage Disease’, ‘GSD’ and specific subtypes/eponyms) and (2) Intervention (e.g., ‘Continuous Glucose Monitoring’, ‘CGM’, ‘Flash Glucose Monitoring’). To ensure maximum sensitivity and the capture of all relevant clinical and psychosocial endpoints, no search filters regarding specific outcomes, age, or study design were applied. The full search strategy for all databases, including specific keywords and Boolean operators, is available in Table S1.

### 2.3 Eligibility Criteria

Studies were included if they met the following PICOS criteria:

- Population: Patients of any age diagnosed with any subtype of GSD.
- Intervention: Use of real‐time or blinded CGM or FGM.
- Comparator: SMBG, placebo or no comparator.
- Outcomes: Glycemic metrics, metabolic/secondary outcomes, device accuracy, or psychosocial measures.
- Study Design: RCTs, observational studies, case series and case reports.
- Exclusion: Conference abstracts without full text, reviews, editorials, animal studies and articles not available in English.

### 2.4 Study Selection

**FIGURE 1:**
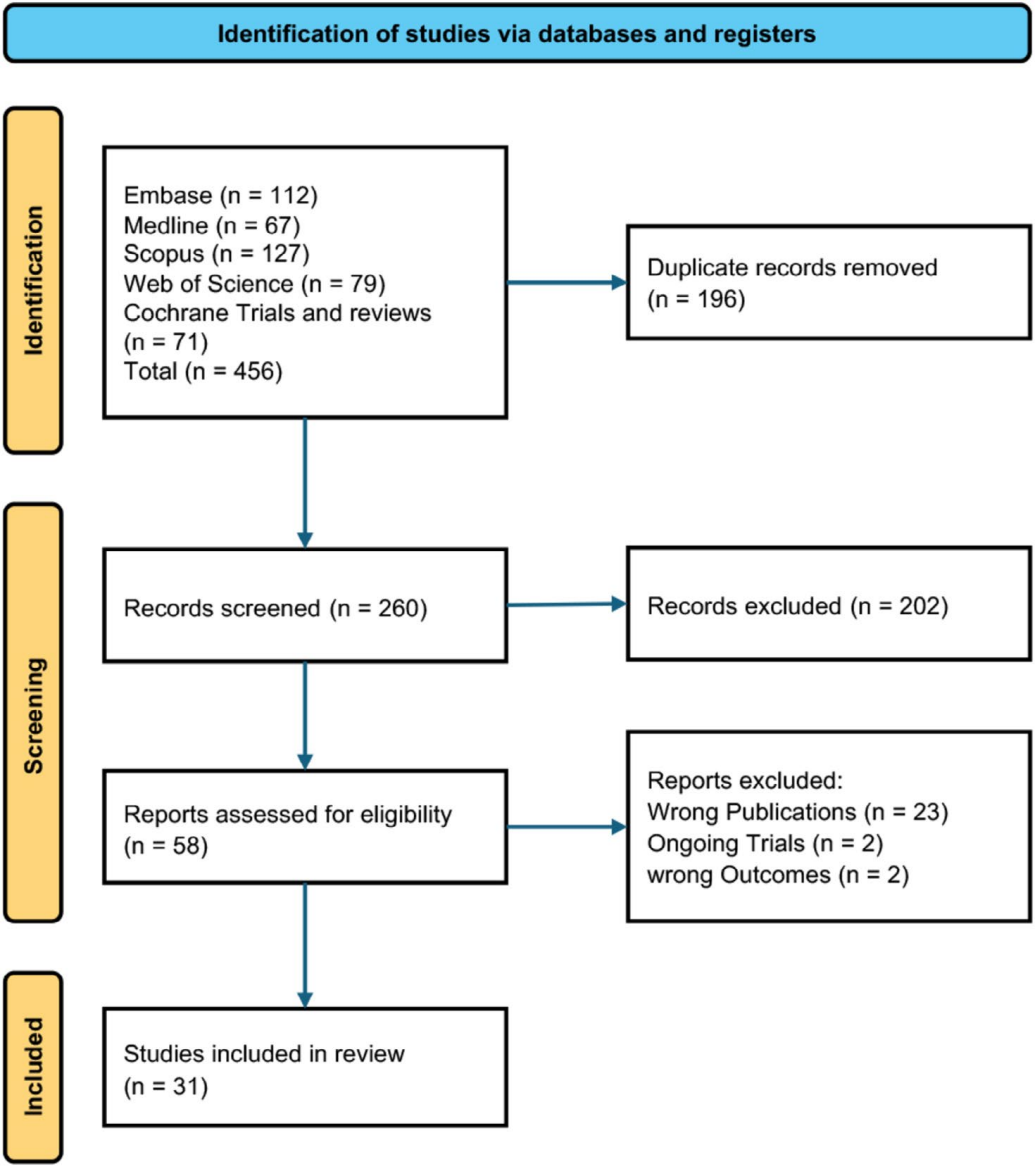
PRISMA 2020 flow diagram depicting the systematic literature search and selection process. A total of 456 records were initially identified; after duplicate removal and screening, 31 studies met the inclusion criteria for the final review.

All identified records were imported into EndNote reference management software for duplicate removal. The screening process was led by KA and conducted in two independent stages. First, two reviewers (KA and MG) independently screened titles and abstracts of all 260 records to identify potentially relevant studies, blinded to each other's decisions. Disagreements at this stage were resolved by discussion with a third reviewer (FA). In the second stage, full texts of candidate articles were assessed independently by KA and RM against eligibility criteria, with final consensus and methodological oversight provided by SA. The selection process is detailed in the PRISMA flow diagram (Figure 1).

### 2.5 Data Extraction and Quality Assessment

Data extraction was performed independently by RM and FA into a standardized Excel pilot form, capturing study characteristics, population demographics, CGM device specifications and key outcomes. To ensure accuracy, KA performed a final cross‐verification of 100% of the extracted data against the original full‐text articles. The methodological quality of included studies was assessed using the Cochrane Risk of Bias 2 (RoB 2) tool for randomized trials, the Risk of Bias in Non‐randomized Studies—of Interventions (ROBINS‐I) tool for single‐arm and observational interventions and the Joanna Briggs Institute (JBI) checklist for case reports and series.

### 2.6 Data Synthesis

Due to the significant heterogeneity in defining euglycemic ranges, the variation in reporting metrics (mean/SD vs. median/IQR) and the predominance of single‐arm observational designs without control groups, a formal quantitative meta‐analysis was not feasible. Therefore, results are presented via narrative synthesis. Extracted data were tabulated and synthesized across five thematic domains: (A) glycemic control metrics; (B) hypoglycemia detection and safety; (C) glycemic variability; (D) secondary metabolic outcomes; and (E) clinical and psychosocial impact. Glucose values reported in mmol/L were converted to mg/dL for consistency.

## 3 Results

### 3.1 Study Selection and Characteristics

The systematic search identified a total of 456 records across five databases (PubMed *n* = 67, Embase *n* = 112, Scopus *n* = 127, Web of Science *n* = 79, Cochrane *n* = 71). Following duplicate removal and title/abstract screening, 58 articles were assessed for full‐text eligibility. Of these, 27 were excluded, primarily due to being conference abstracts without sufficient data (*n* = 18), review articles (*n* = 5), ongoing trials (*n* = 2) or reporting irrelevant outcomes (*n* = 2). Ultimately, 31 studies met all inclusion criteria (Figure 1). The included studies encompassed a heterogeneous mix of observational designs, case reports/series (*n* = 13), one randomized controlled trial (RCT) and one gene therapy trial, with detailed characteristics summarized in Table 1.

**TABLE 1:** Characteristics of included studies (*N* = 31).

Study ID	Study design	Population	CGM device	Comparator/intervention	Main outcome domian	Quality rating
Peeks et al. [21] the Netherlands	Retrospective Observational	*N* = 15 GSD Ia, Ib, IIIa… Age: 2–22 years	Dexcom G4/G6	Comparator: Multiple (Gastric Drip vs. UCCS; UCCS vs. Glycosade)	Glycemic Control	Fair
Rousseau et al. [22] Canada	Prospective Cohort	*N* = 9 GSD Ia Age: Mean 29.9 years	Medtronic iPro2	Comparator: Standard UCCS vs. Glycosade	Sleep and Psychosocial Outcomes	Fair
White et al. [6] United Kingdom	Descriptive Cohort	*N* = 23 GSD 0, Ia, Ib, III… Age: 7 m—20y	Medtronic iPro2	Comparator: Historical profiles vs. Home CGM	Hypoglycemia Detection and Prevention	Poor
Akin et al. [23] Turkey	Descriptive Cohort	*N* = 12 GSD Ia, III Age: Median 7.7	Medtronic iPro2	Observational: Standard Therapy (UCCS/Glycosade)	Glycemic Control and Nutritional Outcomes	Fair
Herbert et al. [7] USA	Descriptive Cohort	*N* = 20 GSD Ia, Ib, IIIa… Age: Median 13	Dexcom G4/G6	Comparator: CGM vs. SMBG	Hypoglycemia Detection and Patient Education	Fair
Fukuda et al. [24] Japan	Cross‐Sectional	*N* = 30 GSD Ia Age: 3–52 years	Dexcom G4/G6	Intervention: High‐carb diet optimization	Hypoglycemia Burden and Glycemic Variability	Good
Overduin et al. [25] the Netherlands	Retrospective Observational	*N* = 61 GSD Ia, Ib, III, VI… Age: Mixed (Ped/Adult)	Dexcom G4/G6	Comparator: Healthy Controls	Glycemic Control and Metrics	Good
Kasapkara et al. [26] Turkey	Prospective Cohort	*N* = 16 GSD Ia, Ib Age: 2–18 years	Medtronic iPro2	Comparator: Pre‐ vs. Post‐diet revision	Hypoglycemia Detection and Prevention	Fair
Rossi et al. [8] the Netherlands	Prospective Observational	*N* = 10 (+10 Controls) GSD Ia Age: Median 22.2 years	Dexcom G4/G6	Comparator: Healthy Volunteers	Glycemic Control and Metrics	Good
Kaiser et al. [27] Switzerland	Registry Analysis	*N* = 25 GSD Ia, Ib Age: Median 20 years	FreeStyle Libre	Observational: Correlation with complications	Hypoglycemia Burden and Metabolic Associations	Good
Gupta et al. [28] India	Pre‐Post Intervention	*N* = 20 (10 completed) GSD I, III, VI, IX… Age: Median 3.68 years	Medtronic iPro2	Comparator: Baseline vs. Post‐counselling	Glycemic Control and Metrics	Fair
Hochuli et al. [29] Switzerland	Randomized Crossover	*N* = 5 GSD Ia, Ib Age: 19–35 years	CGM (Various)	Comparator: UCCS vs. Pasta vs. Glycosade	Hypoglycemia Prevention	Good
Hsu et al. [30] Taiwan	Pre‐Post Intervention	*N* = 9 GSD Ia Age: 9–33 years	Medtronic iPro2	Comparator: UCCS vs. Glycosade	Sleep and Psychosocial Outcomes	Fair
Massimino et al. [31] Italy	Case Report	*N* = 1 GSD IIIa Age: 24 years	FreeStyle Libre	Comparator: Baseline vs. Diet Change	Glycemic Control and Metrics	Poor
Agnoletto et al. [32] Australia	Prospective Pilot	*N* = 7 GSD Ia, Ib, VI, IX… Age: 1–17 years	Dexcom G4/G6	Observational: Nocturnal strategies comparison	Sleep Quality	Fair
Maran et al. [33] Italy	Descriptive	*N* = 6 GSD Ia, Ib, III Age: 11–47 years	Glucoday	Comparator: CGM vs. SMBG correlation	Hypoglycemia Detection	Fair
Marcalo et al. [34] Portugal	Case Report	*N* = 1 GSD Ia Age: 22 years	FreeStyle Libre	Comparator: Pre‐ vs. Post‐FGM	Glycemic Control and Metrics	Poor
Amuedo et al. [35] Spain	Longitudinal	*N* = 18 (5 GSD) GSD Ib, IIIa, IX… Age: 30.5 ± 12.5	FreeStyle Libre	Comparator: Baseline vs. 2‐month follow‐up	Hypoglycemia Prevention	Poor
Ward et al. [36] USA	Case Report	*N* = 1 GSD Ia Age: 10 months	Dexcom G4/G6	Intervention: Transition to Gastrostomy	Hypoglycemia Prevention	Poor
Fu et al. [37] China	Case Report	*N* = 1 GSD 0a Age: 4 years	Medtronic iPro2	Comparator: Before vs. After diet	Hypoglycemia Detection	Poor
Sanli et al. [38] Turkey	Prospective	*N* = 7 GSD Ia, Ib Age: Mean 9.6 years	Medtronic iPro2	Comparator: Baseline vs. Fibre Supplement	Metabolic and Nutritional Outcomes	Poor
Yatabe et al. [39] Japan	Case Report	*N* = 1 GSD I Age: 57 years	CGM (Various)	Intervention: Artificial Pancreas	Perioperative Glucose Control	Poor
Weinstein et al. [40] Multicenter (USA/EU)	Phase 1/2 Trial	*N* = 12 GSD Ia Age: 18–57 years	CGM (Various)	Comparator: Baseline vs. Gene Therapy	Glycemic Control and Metrics	Fair
Chen et al. [41] China	Case Report	*N* = 1 GSD XI (Fanconi‐Bickel) Age: 6 months	CGM (Various)	Intervention: Diagnosis of dysglycemia	Diagnostic Utility	Poor
Bahíllo‐Curieses [42] Spain	Case Report	*N* = 1 GSD XI Age: Neonate (19 days)	Medtronic iPro2	Intervention: Neonatal management	Diagnostic Utility	Poor
Korljan et al. [43] Croatia	Case Report	*N* = 1 GSD Ia Age: 23 years	Medtronic iPro2	Intervention: Obesity management	Hypoglycemia Detection and Prevention	Poor
Bonnet et al. [44] France	Case Report	*N* = 1 GSD IIIa Age: 32 years	FreeStyle Libre	Intervention: Pregnancy management	Hypoglycemia Prevention	Poor
Kanemaru et al. [45] Japan	Case Report	*N* = 1 GSD Ia Age: 59 years	FreeStyle Libre	Intervention: GSD + Diabetes management	Glycemic Control and Metrics	Poor
Hershkovitz et al. [46] Israel	Case Series	*N* = 4 GSD Ia Age: 2–15 years	CGM (Various)	Intervention: Early CGM validation	Hypoglycemia Detection	Fair
Teufel‐Schäfer [47] Germany	Case Report	*N* = 1 GSD IXb Age: 8 years	Dexcom G4/G6	Outcome: Adverse Event (Dermatitis)	Device Tolerance and Feasibility	Poor
Alkundi et al. [48] UK	Case Report	*N* = 1 GSD Ia Age: 27 years	CGM (Various)	Outcome: Hypoglycemia Unawareness	Hypoglycemia Detection	Poor

*Note:* ‘Prospective Cohort’ refers to longitudinal follow‐up; ‘Descriptive Cohort’ refers to cross‐sectional or single‐arm observational data without a strict prospective protocol. Methodological quality was assessed using the ROBINS‐I tool for observational/interventional studies and JBI checklists for case reports. Ratings were categorized as Good (Low risk of bias, robust methodology), Fair (Moderate risk of bias, minor methodological limitations), or Poor (High risk of bias, significant limitations or single case reports).

Abbreviations: CGM, Continuous Glucose Monitoring; FGM, Flash Glucose Monitoring; GSD, Glycogen Storage Disease; IQR, Interquartile Range; *N*, Number of participants; SD, Standard Deviation; SMBG, Self‐Monitoring of Blood Glucose; UCCS, Uncooked Cornstarch.

### 3.2 Risk of Bias and Quality Assessment

The overall methodological quality of the included studies was heterogeneous, reflecting the rarity of the condition and the predominance of observational designs. Using the ROBINS‐I tool and JBI checklists, 5 studies were rated as Good quality, characterized by robust sample sizes relative to disease prevalence and the inclusion of control groups or rigorous registry data (Fukuda et al., Overduin et al., Rossi et al., Kaiser et al., Hochuli et al.). 11 studies were classified as Fair quality; this category included the majority of prospective cohorts and the gene therapy trial (Weinstein et al.), which generally employed sound methodology but lacked external control groups or blinding. The remaining 15 studies were rated as Poor quality. This group primarily consisted of single‐patient case reports and small case series (*n* < 10) with significant selection bias, lack of standardized outcomes and retrospective designs that limited the ability to draw causal inferences about CGM efficacy. The overall quality of the included studies is included in Table 1.

### 3.3 Study Locations and Populations

**FIGURE 2:**
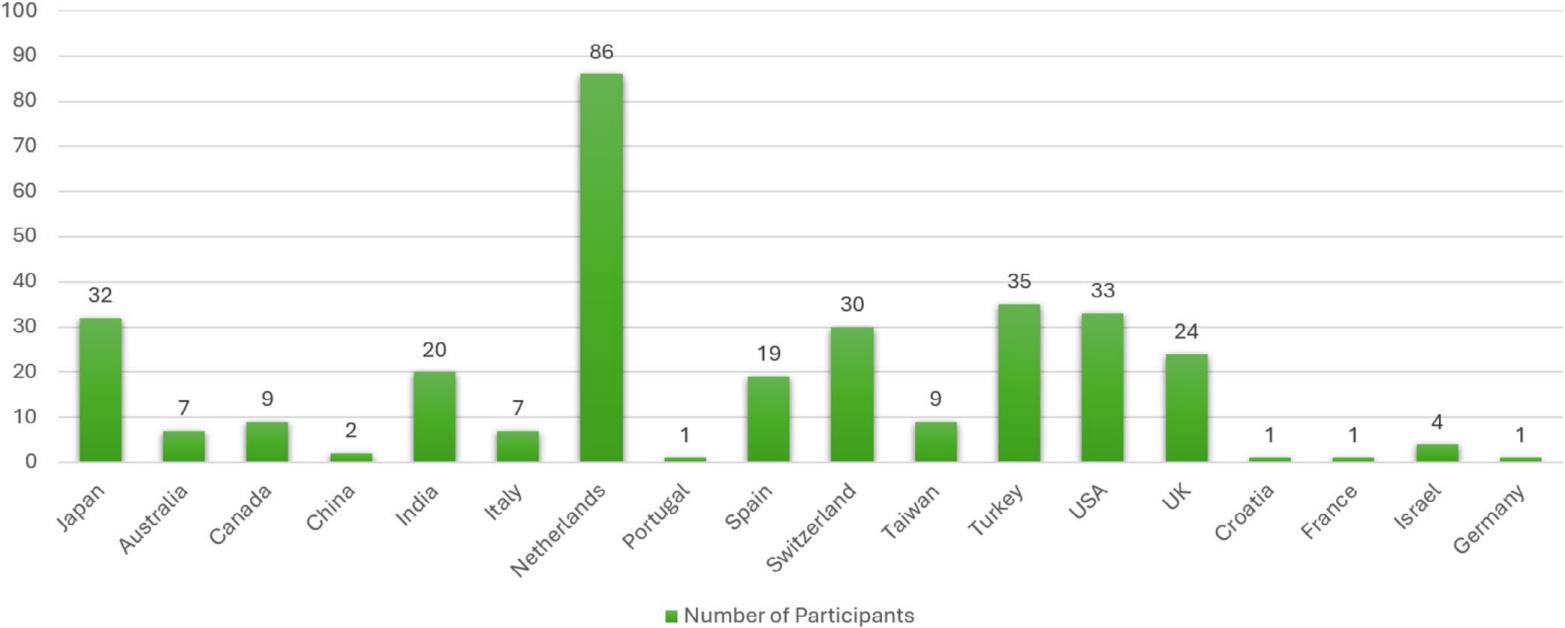
Geographic distribution of study participants. The bar chart illustrates the total number of participants (*N* = 323) contributed by each country across the included studies, highlighting significant contributions from the Netherlands (*n* = 86), Turkey (*n* = 35) and the USA (*n* = 33).

The geographic distribution of the included studies is diverse, covering Asia, Europe, North America and the Middle East. The highest number of participants was reported in studies from the Netherlands (*n* = 86), followed by Turkey (*n* = 35) and the USA (*n* = 33). Japan contributed a significant number of participants (*n* = 32), driven by multiple case reports and small cohorts. Other contributing countries are detailed in Figure 2.

### 3.4 Participant Characteristics

There were 323 participants in all 31 investigations. The study population was mostly paediatric, from neonates (19 days) to adults (57 years). Most studies focused on GSD Type I (Ia and Ib). However, data were available for GSD Type III, VI/IX, 0, IV and XI (Fanconi‐Bickel Syndrome).

The included studies reflect a wide range of research types and methodological approaches. Observational studies provide much of the evidence. Single‐center retrospective analyses [21, 25] and early CGM evaluations [46] use clinical data. Additionally, prospective cohort studies [8, 22, 26] and a pilot study [32], collect longitudinal data. Two studies used cross‐sectional methodology [24, 27].

This review features a range of interventional studies; a longitudinal quasi‐experimental study [35], an open‐label prospective study [38] and a Phase 1/2 gene therapy trial utilizing CGM as a long‐term efficacy endpoint [40]. Several studies examined targeted nutritional strategies, such as the transition to extended‐release cornstarch or modified feeding schedules [28, 29, 30]. Finally, descriptive studies characterize patient populations or experiences [6, 7, 23, 33]. Case reports include detailed descriptions of specific patients, providing hypothesis‐generating insights into unusual clinical presentations such as pregnancy [44], perioperative management [39], diabetes comorbidity [45] and therapeutic strategies [31, 34, 36, 37, 41, 42, 43, 47].

### 3.5 CGM Technology and Implementation

Multiple CGM systems were used in the studies. Dexcom G4/G6 [7, 8, 21, 24, 25, 32, 36, 40, 44, 47] and FreeStyle Libre flash glucose monitoring systems dominated real‐time devices. Blinded or retrospective professional monitoring was mostly done with Medtronic iPro2/Enlite systems [22, 23, 26, 27, 28, 29, 37, 38, 43, 47], however, the CGMS Gold [6] and Glucoday microdialysis system [33] were discussed before. In cross‐sectional studies, wear lasts 3–7 days, while gene therapy and pregnancy protocols require weeks–months of monitoring [40, 44]. One longitudinal observational study tracked a sample of participants for 157 days [21].

### 3.6 Blinding and Calibration

In 16 studies, glucose data was unblinded or real‐time, allowing clinical decisions like real‐time alerts for hypoglycemia unawareness [48] and artificial pancreas systems [39], while 10 studies used blinded [22, 23, 26, 27, 32, 43, 46] or retrospective protocols [6, 28, 37, 46] mainly with Medtronic iPro2 systems. Two studies [21, 42] had mixed status, and several were unreported. 14 Medtronic, Dexcom 4 and Glucoday studies used capillary glucose 1–5 times daily, with Glucoday needing a single venous glucose test at least 2 h after catheter installation for 24‐h monitoring [33]. Yatabe et al.'s artificial pancreas STG‐55 was calibrated every 4 h [39]. 11 studies implemented CGM devices that were factory‐calibrated (e.g., Dexcom G6, FreeStyle Libre FGM which requires no user calibration), mixed in 2 studies [21, 42] and not explicitly mentioned in 7 studies [37, 38, 40, 41, 42, 47, 48].

### 3.7 Primary Outcome: Impact on Glycemic Control

#### 3.7.1 Mean Glucose and Time in Range (TIR)

As summarized in Table 2, the included studies reported heterogeneous but clinically meaningful CGM‐based glycemic and metabolic outcomes, including TIR/TBR metrics, hypoglycemia detection and secondary metabolic effects. CGM enabled continuous glycemic stability quantification, notably using the Time in Range (TIR) metric, which 11 of 21 studies reported. However, procedures defined ‘euglycemia’ and target range differently, preventing a meta‐analysis. GSD requires rigorous management to prevent counter‐regulatory reactions; hence, Rossi et al. [8] and Overduin et al. [25] used a strict therapeutic target of 70–140 mg/dL. Other research, like Amuedo et al. [35] used the diabetes treatment consensus target of 70–180 mg/dL. Researchers like Peeks et al. [21] and Marcalo et al. [34] used both ranges for reporting TIR.

**TABLE 2:** Key clinical and metabolic outcomes of included studies.

Study ID	Glycemic Control and Hypoglycemia (CGM Metrics)	Metabolic and secondary outcomes	Psychosocial, safety and accuracy
Peeks et al. [21] the Netherlands	TIR: 97.5% (UCCS) vs. 73.7% (Gastric Drip). TBR: Reduced variability with UCCS.	Lipids: Triglycerides (9.4 ➔ 5.4 mmol/L) and Cholesterol (6.9 ➔ 6.0 mmol/L) reduced. Liver: Hepatomegaly reduced (26 ➔ 19 cm).	Accuracy: Strong correlation between Dexcom G4 and capillary glucose.
Rousseau et al. [22] Canada	TIR: 76% (4–8 mmol/L). TBR: Significant time < 4 mmol/L detected.	Lipids: Baseline Triglycerides 3.8 mmol/L.	Sleep: PSQI score improved from 6.25 (poor) to 3.5 (normal).
White et al. [6] United Kingdom	TBR: Reduced from 17.5% to 6% (< 3.5 mmol/L) after UCCS optimization (GSD 0).	Ketones: Morning ketones identified overnight fasting intolerance.	Accuracy: Reliability noted to be lower in hypoglycemic range.
Akin et al. [23] Turkey	TIR: Mean 85.6% (70–150 mg/dL). TBR: Median 16.0% (< 70 mg/dL).	Liver: AST/ALT and Triglycerides significantly higher in GSD III vs. GSD Ia.	Accuracy: Mean Absolute Difference (MAD) < 18% (Good).
Herbert et al. [7] USA	TIR: Mean 90.3% (70–150 mg/dL). TBR: GSD I spent 8.4% time < 70 mg/dL.	Lipids: Triglycerides reduced following dietary adjustment (Case #3).	Accuracy: Strong correlation (*r* ^2^ = 0.57), consistent in hypoglycemia.
Fukuda et al. [24] Japan	TBR: Hypoglycemia duration (< 70 mg/dL) increased with age (Children: 80 min– > Adults: 132 min/day).	Complications: Hypertriglyceridemia (90%) and Hyperuricemia (83%) prevalence high.	Accuracy: MARD 13.0% (High).
Overduin et al. [25] the Netherlands	TIR: GSD Ia (88%) and Ib (90%) significantly lower than healthy controls (96%).	Growth: Failure to thrive noted in GSD IIIa case.	Safety: Dexcom G6 has high sensitivity for hypoglycemia.
Kasapkara et al. [26] Turkey	TBR: Reduced from 7.06% to 2.25% (*p* = 0.002). Hypo: Asymptomatic nocturnal hypoglycemia detected.	Lipids: Triglycerides reduced (574 ➔ 457 mg/dL). Lactate: Reduced (3.8 ➔ 3.1 mmol/L). Liver: Size reduced (8.2 ➔ 6.3 cm).	Accuracy: Correlation *r* = 0.86 (High). PROs: Reduced aversion to fingersticks.
Rossi et al. [8] the Netherlands	TIR: GSD Ia (82.6%) lower than healthy controls (92.6%).	Bias: Non‐significant overestimation (+0.85 mmol/L) by CGM.	Safety: Prevented Level 3 hypoglycemia events.
Kaiser et al. [27] Switzerland	TBR: Median 2.2 h/day < 4 mmol/L. Hypo: 44% of patients were asymptomatic.	Complications: Adenomas associated with higher frequency/AUC of hypoglycemia.	QoL: SF‐12 scores in normal range.
Gupta et al. [28] India	TIR: Improved from 87.2% to 96.4% (*p* = 0.006). TBR: Reduced from 10.1% to 1.8% (*p* = 0.006).	Lipids: Substantial triglyceride decline in one patient (1130 ➔ 410 mg/dL).	Accuracy: CGM fails to detect absolute glucose < 40 mg/dL.
Hochuli et al. [29] Switzerland	TBR: No nocturnal hypoglycemia with UCCS, Pasta or Modified Starch.	Lipids: Triglycerides 3.4–7.1 mmol/L.	Preferences: Pasta meal preferred as ‘more palatable.’
Hsu et al. [30] Taiwan	Mean Glucose: Morning glucose improved (80– > 86.5 mg/dL).	Liver: AST (78 ➔ 37 U/L) and ALT (69 ➔ 41 U/L) reduced (*p* = 0.013).	Sleep: PSQI score improved (5.8– > 3.0) in adults.
Massimino et al. [31] Italy	TIR: Improved from 91% to 96% after 24 months.	Metabolic: Insulin (41 ➔ 12 μU/mL) and Lactate (5.7 ➔ 0.9) normalized.	PROs: Patient reported ‘greater exercise capacity.’
Agnoletto et al. [32] Australia	TBR: One participant recorded 10.6% time < 3.5 mmol/L.	Sleep: Poor sleep quality linked to hypoglycemia.	PROs: Confirmed subjective sleep quality issues.
Maran et al. [33] Italy	Hypo: Unrecognized hypoglycemia (< 60 mg/dL) found in 4/6 patients.	Metabolic: Hyperuricemia and hypertension noted.	Accuracy: Mean absolute difference < 2% (Excellent).
Marcalo et al. [34] Portugal	TIR: Improved from 61% to 82% over 1 year.	Lipids: Triglycerides (396 ➔ 261 mg/dL) improved.	Safety: FGM useful but requires confirmatory fingerstick.
Amuedo et al. [35] Spain	TIR: Improved from 98% to 99% (*p* = 0.020).	Status: HbA1c 5.5%. BMI 28.1 kg/m^2^.	PROs: Improved Quality of Life perception.
Ward et al. [36] USA	Hypo: Detected recurrent severe hypoglycemia (22 mg/dL).	Status: Stabilized after transition to gastrostomy.	PROs: Reduced ‘severe family distress.’
Fu et al. [37] China	TBR: Hypoglycemia frequency reduced from 25% to 12%.	Lactate: Normalized (4.64 ➔ 1.34 mmol/L).	PROs: Maintained good mental status.
Sanli et al. [38] Turkey	Hypo: All detected events were asymptomatic.	Lipids: Triglycerides reduced (610 ➔ 393 mg/dL) with fibre.	Safety: No GI side effects reported.
Yatabe et al. [39] Japan	Safety: No intraoperative hypoglycemia occurred (Artificial Pancreas).	Lactate: Transient intraoperative increase, normalized in ICU.	Qualitative: Reduced workload for anesthesiologists.
Weinstein et al. [40] Multicenter (USA/EU)	TIR: Increased from 72% to 86% (Cohort 3). TBR: Nocturnal hypoglycemia rare post‐treatment.	Lipids: Mean triglyceride increase (287 mg/dL) observed.	PROs: Patient Global Impression of Change (PGIC) validated improvement.
Chen et al. [41] China	TBR: Documented pre‐prandial hypoglycemia pattern.	Status: Severe hypokalemia and rickets noted.	Qualitative: Identified ‘hypo‐hyper’ pattern of Fanconi‐Bickel.
Bahíllo‐Curieses [42] Spain	Hypo: Recurrent fasting hypoglycemia confirmed.	Status: High Alkaline Phosphatase; Adequate weight gain.	Qualitative: Trend arrows helped avoid ‘rebound hyperglycemia.’
Korljan et al. [43] Croatia	TBR: 0% (No hypoglycemia).	Lipids: Triglycerides (2.0 ➔ 1.7 mmol/L) improved with weight loss.	Qualitative: Essential for safe weight loss in obese GSD Ia.
Bonnet et al. [44] France	Hypo: Nocturnal episodes markedly reduced.	Pregnancy: Uncomplicated course; healthy term delivery.	Qualitative: ‘Peace of mind’ during high‐risk pregnancy.
Kanemaru et al. [45] Japan	TBR: 1% time < 70 mg/dL.	Lipids: Triglycerides improved (287 ➔ 192 mg/dL).	Qualitative: Managed ‘double burden’ of GSD + Diabetes.
Hershkovitz et al. [46] Israel	Hypo: Detected in 100% of patients (1%–10% of time).	Status: Dyslipidemia and growth retardation present.	Accuracy: High correlation (*r* ≥ 0.9).
Teufel‐Schäfer [47] Germany	N/A (Discontinued)	N/A	Safety: Severe allergic contact dermatitis caused discontinuation.
Alkundi et al. [48] UK	TBR: 12% time < 4 mmol/L (Hypoglycemia Unawareness).	Lactate: Improved from 17.2 to 6.2 mmol/L.	Qualitative: Alerts patients before symptoms manifest.

*Note:* Percentages for TIR/TBR/TAR reflect the proportion of CGM wear time spent within the specified glucose thresholds, which varied across studies (e.g., 60–120, 70–150 or 70–180 mg/dL) and therefore are not directly comparable across all rows. Glycemic and metabolic outcomes are reported in the original units used by each study (mg/dL or mmol/L) and summarized descriptively due to heterogeneity in outcome definitions and reporting formats.

Abbreviations: AST/ALT/GGT, aspartate aminotransferase/alanine aminotransferase/gamma‐glutamyl transferase; AUC, area under the curve; BOHB, beta‐hydroxybutyrate; CGM, continuous glucose monitoring; FGM/isCGM, (intermittently scanned) flash glucose monitoring; HbA1c, glycated haemoglobin; MARD/MAD, mean absolute relative/absolute difference; PGIC/PGIS, Patient Global Impression of Change/Severity; PROs, patient‐reported outcomes; PSQI, Pittsburgh Sleep Quality Index; QoL, quality of life; rCGM, real‐time CGM; SF‐12, 12‐Item Short Form Health Survey; TAR, time above range; TBR, time below range; TIR, time in range.

Drawing on higher‐quality observational data, Overduin et al. [25] demonstrated that GSD Ia, Ib and III patients spent significantly less time (88.1%, 90.0% and 91.1%, respectively) in the strict euglycemic range (70–140 mg/dL) compared to age‐matched healthy controls (95.8%). In interventional settings, well‐designed studies underscored that CGM accurately tracks progress in metabolic optimization. Notably, in the first gene therapy trial for GSD Ia, Weinstein et al. [40] utilized CGM as a primary effectiveness objective, recording a robust improvement in euglycemia (60–120 mg/dL) from a baseline of 72%–86% 1 year post‐treatment. Similarly, Gupta et al. [28] provided valuable evidence that CGM‐informed dietary adjustments significantly increased median TIR (70–150 mg/dL) from 87.2% to 96.4% *p* = 0.006 suggesting that identifying asymptomatic fluctuations optimizes control better than spot testing alone. These pivotal findings are further corroborated by lower‐quality observational reports; for instance, Massimino et al. [31] noted that a stepwise carbohydrate reduction enhanced TIR from 91% to 96% over 24 months, aligning with the broader evidence base.

To identify safety signals that spot testing often misses, higher‐quality comparative studies emphasize the necessity of CGM beyond merely tracking average control. Providing robust evidence, Rossi et al. [8] demonstrated that even in treated cohorts, adult GSD Ia patients experienced significantly more hypoglycemia (Time Below Range (TBR) < 70 mg/dL: 3.4% vs. 0.7% in healthy controls), with 60% experiencing level 2 hypoglycemia (< 54 mg/dL) during 24‐h monitoring. Supporting this, moderately graded observational data by Hershkovitz et al. [46] revealed that children spent up to 10.29% of the day in severe hypoglycemia (< 50 mg/dL). Furthermore, Kasapkara et al. [26] highlighted the clinical utility of CGM, showing that sensor‐guided dietary changes significantly reduced hypoglycemia time (< 70 mg/dL) from 7.06% to 2.25% post‐intervention (*p* = 0.002). These systematic findings are echoed by lower‐quality data and individual reports; for instance, White et al. [6] and Alkundi et al. [48] described recurrent asymptomatic hypoglycemic episodes, including a TBR (< 72 mg/dL) of 12% in one adult, that capillary tests failed to detect.

Conversely, an overzealous focus on preventing hypoglycemia, often resulting in overtreatment with complex carbohydrates, can inadvertently lead to chronic hyperglycemia and increase the risk of metabolic complications such as insulin resistance. The clinical objective remains maintaining true euglycemia, rather than merely avoiding low glucose levels. Highlighting the prevalence of this overtreatment risk through comparative data, Rossi et al. [8] found that GSD Ia patients spent more time above range (> 140 mg/dL) than matched controls (13.5% vs. 6.4%). Similarly, Herbert et al. [7] reported daytime and nocturnal hyperglycemic excursions, which are typically missed without continuous monitoring, further underscoring the consequences of over‐supplementation. Corroborating these broader findings on an individual level, Kanemaru et al. [45] detailed a case report of a GSD Ia patient with co‐occurring diabetes who spent 35% of the time in hyperglycemia (> 180 mg/dL) until CGM‐guided modifications successfully reduced it to 6%.

#### 3.7.2 Glycemic Variability

Beyond mean glucose levels, CGM provides critical data on glycemic variability (GV), a key marker of metabolic stability in GSD that traditional intermittent testing often fails to capture. The Coefficient of Variation (CV) and Standard Deviation (SD) are the primary metrics used to quantify these fluctuations. In the large retrospective cohort study by Overduin et al. [25] demonstrated that patients with GSD Ia (> 6 years old) exhibited a median glucose CV of 20.3% (IQR 18.8%–23.4%), which was significantly higher than the reference values for healthy individuals. This finding was prospectively validated by Rossi et al. [8], who compared 10 adult GSD Ia patients directly with matched healthy volunteers; they found that patients had a significantly higher mean 24‐h glucose SD (27 vs. 16 mg/dL, *p* < 0.05) and CV, confirming that even well‐treated patients experience greater glycemic instability than the general population. Fukuda et al. [24] found a mean glucose CV of 23.0% ± 5.9% in a cross‐sectional study of 30 Japanese patients with GSD Ia, highlighting the difficulty of maintaining stable euglycemia.

Variability metrics also differ across GSD subtypes. Overduin et al. [25] noted that while GSD Ia and Ib patients had high variability with reduced TIR, GSD IX patients demonstrated CGM measures closer to healthy controls, typically showing only modestly increased time above range. This aligns with broader clinical data classifying GSD IX among the milder hepatic GSDs, wherein metabolic and glycemic homeostasis are generally more responsive to and easily normalized by nutritional treatments compared to GSD I [49, 50, 51]. Furthermore, specific interventions influence overall glycemic stability. Peeks et al. [21] highlighted how switching from continuous gastric drip feeding to uncooked cornstarch reduced hypoglycemia time and stabilized glucose profiles in individual cases. Rousseau et al. [22] used CGM to show that Glycosade allowed patients to maintain euglycemia for 8.0 h (median 8.0 h vs. 4.0 h with conventional cornstarch) while keeping time above 144 mg/dL at 0.05. While these intervention findings primarily emphasize the extension of euglycemia and the prevention of hypoglycemic excursions rather than reporting standardized GV metrics, the mitigation of rapid glucose declines and subsequent overtreatment inherently contributes to a more stable overall glycemic profile.

#### 3.7.3 CGM Accuracy and Reliability

While CGM is well‐established in diabetes management, its adoption in GSD depends critically on its accuracy in the hypoglycemic range, where missed events can be life‐threatening. The literature reveals a crucial distinction between correlation (trend accuracy) and bias (absolute safety).

##### 3.7.3.1 Correlation and Trend Accuracy

Multiple studies confirm that interstitial CGM readings correlate strongly with capillary blood glucose (SMBG) across the euglycemic and hyperglycemic ranges. Drawing on moderately graded observational data, several studies have established high accuracy metrics. Early validation by Hershkovitz et al. [46] demonstrated a high correlation coefficient (*r* ≥ 0.9) in paediatric patients. Corroborating this in a pivotal study of 20 patients (GSD I, III, IX), Herbert et al. [7] reported a strong positive correlation (*r* ^2^ = 0.57, *p* < 0.0001) that remained consistent even within the hypoglycemic range (< 70 mg/dL, *r* ^2^ = 0.53). Similarly, in a paediatric GSD I cohort, Kasapkara et al. [26] found a high correlation between CGM and glucometer results (*r* ^2^ = 0.86). Further supporting these findings, Maran et al. [33] noted an excellent correlation (*r* = 0.9) using microdialysis‐based systems, with a mean absolute difference of less than 2% in the hypoglycemic range, while Akin et al. [23] recently reported a Mean Absolute Difference (MAD) of < 18% for GSD patients, meeting standard clinical accuracy criteria. Finally, these consistent trends are supplemented by lower‐quality, individual‐level evidence; for instance, Yatabe et al. [39] noted near‐identical readings between an artificial pancreas sensor (86 mg/dL) and a central laboratory reference (87 mg/dL) after surgery in critical care.

##### 3.7.3.2 Systematic Bias and Safety Concerns

Despite these strong correlations, systematic bias remains a safety risk, particularly at the lower limits of detection. Rossi et al. [8] performed a Bland–Altman analysis on adult GSD Ia patients and identified a mean positive bias of +15.3 mg/dL, which increased to +19.5 mg/dL during hypoglycemia. This tendency for CGM to overestimate glucose levels means that a sensor reading of 63 mg/dL could disguise an actual capillary glucose of 45 mg/dL. Thus, while CGM is a valuable tool for trend analysis and pattern recognition, studies agree that it cannot yet replace confirmatory fingerstick testing. From a clinical safety perspective, capillary testing remains mandatory in two specific scenarios: (1) whenever the CGM reading approaches or falls below the hypoglycemic threshold and (2) whenever a patient experiences symptoms of hypoglycemia, regardless of an ostensibly ‘normal’ sensor display.

#### 3.7.4 Quantitative Summary of Glycemic Outcomes

Despite the heterogeneity in outcome definitions (with target ranges varying across studies, such as 60–120, 70–150 or 70–180 mg/dL), a quantitative synthesis reveals consistent directional benefits. Initially, CGM identified significant unrecognized hypoglycemic events, with adult cohorts spending up to 12% of their monitored time below target ranges (e.g., < 70 mg/dL) despite normal intermittent spot checks. Importantly, following CGM‐guided interventions, several studies (including Peeks et al. [21] and Gupta et al. [28]) reported near‐complete resolution of hypoglycemia, reducing Time Below Range (TBR) to < 1%. Concurrently, Time in Range (TIR) demonstrated substantial improvements, with absolute increases reported from a baseline of 72% up to 86% in recent trials. These quantifiable ranges clearly underscore the clinical magnitude of CGM utility in mitigating severe neuroglycopenia, although standardizing glucose thresholds remains a prerequisite for future direct meta‐analyses.

### 3.8 Secondary Outcomes

**FIGURE 3:**
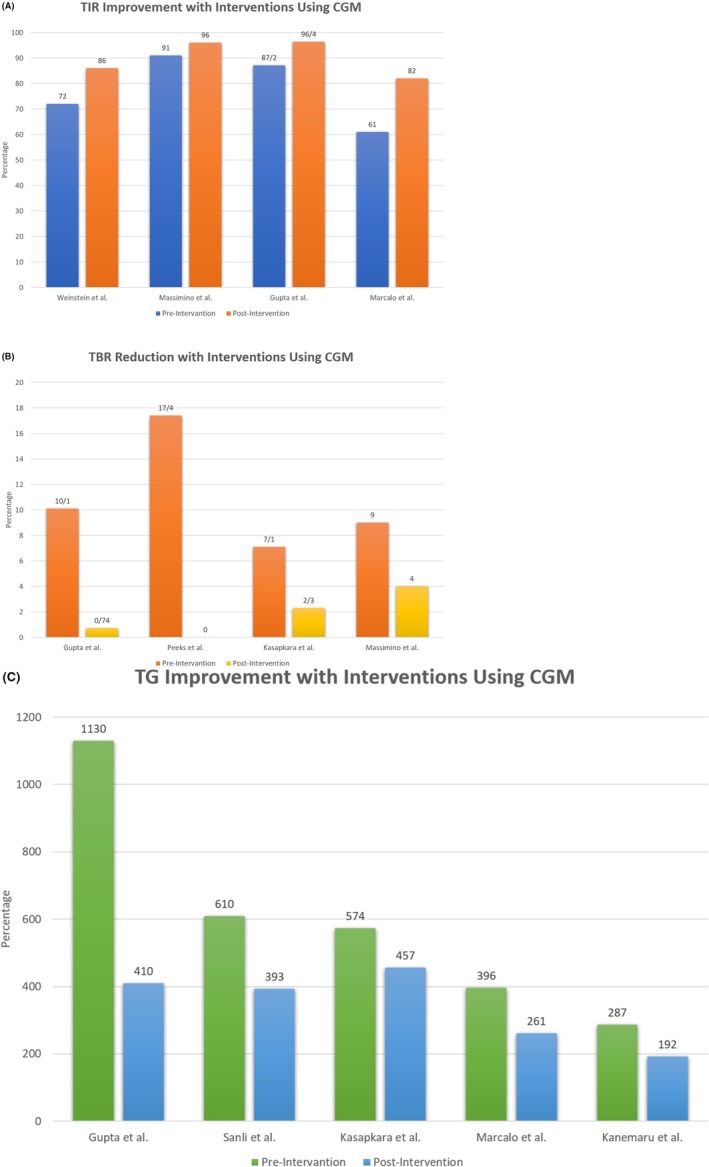
Clinical utility of Continuous Glucose Monitoring (CGM) in Glycogen Storage Diseases. (A) Improvement in Time in Range (TIR). Shifts in normoglycemia following CGM‐guided dietary titration. Bars represent the absolute improvement from baseline (grey) to post‐intervention (blue) in both cohorts and complex case reports (*N* = 36). (B) Reduction in Hypoglycemia (Time Below Range). Comparative analysis showing significant decreases in Time Below Range (< 70 mg/dL) following management optimization. Peeks et al. (Subset I) and Gupta et al. demonstrated near‐complete resolution of hypoglycemia (TBR < 1%) by identifying and correcting overtreatment. (C) Metabolic Impact (Triglycerides). Reductions in serum triglycerides associated with improved glycemic stability. While Sanli et al. (*N* = 11) and Kasapkara et al. (*N* = 14) show statistically significant mean reductions in cohorts, Gupta et al. (Patient #5) illustrates the potential for dramatic lipid normalization (1130 to 410 mg/dL) when ‘hidden’ hypoglycemia is eliminated. Abbreviations: TIR, Time in Range; TBR, Time Below Range; TG, triglycerides; CGM, continuous glucose monitoring.

Long‐term metabolic health shows CGM's clinical value beyond glucose measurements. Figure 3 shows how CGM data enabled proactive management, which improved Time in Range (Figure 3A) and nearly eliminated asymptomatic hypoglycemia (Figure 3B). Importantly, glycemic stabilization directly improved secondary metabolic outcomes, including serum triglycerides (Figure 3C).

#### 3.8.1 Metabolic Control

The clinical utility of CGM extends beyond glycemic observation by guiding dietary and therapeutic interventions that lead to the tangible improvement of secondary metabolic markers. Kasapkara et al. [26] provided the most direct evidence of this link in a paediatric GSD I cohort; following CGM‐guided dietary revisions, patients achieved statistically significant reductions in blood lactate (3.8–3.1 mmol/L, *p* = 0.010) and triglycerides (574–457 mg/dL, *p* = 0.049), which corresponded to a statistically significant decrease in palpable liver size (8.2–6.3 cm, *p* < 0.001). Similarly, Hsu et al. [30] utilized CGM as a safety and monitoring tool for patients transitioning to extended‐release cornstarch (Glycosade) and observed an improvement in hepatic stress markers over 24 weeks, with AST decreasing from 78.8 to 37.8 U/L and ALT from 69.3 to 41.1 U/L (*p* = 0.013), although triglyceride levels remained unchanged.

Metabolic targets must be interpreted within the context of the specific GSD subtype. Based on fair‐quality evidence, Akin et al. [23] demonstrated that while glucose control was comparable, GSD III patients exhibited higher liver transaminases (AST 424 vs. 109 U/L) and triglycerides than GSD Ia patients. Euglycemia alone does not equate to disease quiescence in certain subtypes. As emerging biomarker data and systematic reviews suggest, long‐term complications, such as hepatic fibrosis, myopathy and adenomas, show substantial inter‐subtype variability that is not fully explained by day‐to‐day glucose profiles alone [20, 49, 52, 53].

Furthermore, lower‐quality and anecdotal reports suggest CGM may also be useful in evaluating adjunctive therapies and weight management strategies. For instance, Sanli et al. [38] utilized CGM to monitor dietary fibre (partially hydrolyzed guar gum) supplementation in GSD I. While changes in mean glucose were not statistically significant, the intervention safely reduced glycemic fluctuations and led to significant improvements in specific biochemical parameters, such as triglycerides (610–393 mg/dL, *p* = 0.043) and Gamma‐Glutamyl Transferase (GGT) (147.3–133.6 IU/L, *p* = 0.043) in GSD I patients. Furthermore, an individual report by Korljan Jelaska et al. [43] described utilizing CGM to safely guide caloric restriction in an obese GSD Ia patient; this approach prevented hypoglycemia during weight loss and supported metabolic improvements, including reduced triglycerides (2.0–1.7 mmol/L), cholesterol (5.2–4.5 mmol/L) and uric acid (425–380 nmol/L). Collectively, while these findings are largely observational, they suggest that CGM‐guided monitoring safely facilitates the specific therapeutic and dietary interventions required to mitigate GSD's secondary biochemical derangements, particularly hyperlipidemia and hepatic inflammation.

#### 3.8.2 Clinical and Psychosocial Impact

Beyond metabolic metrics, fair‐quality evidence indicates that CGM significantly alleviates GSD's psychosocial burden, particularly sleep disruption and the pervasive fear of hypoglycemia. Rousseau et al. [22] found that establishing a safe fasting interval, verified by CGM, improved the mean Pittsburgh Sleep Quality Index (PSQI) score from 6.25 (poor sleep) to 3.5 (normal sleep) (*p* < 0.05), directly addressing the chronic sleep deprivation in traditional GSD management. Similarly, after a comparable intervention, Hsu et al. [30] found a statistically significant improvement in PSQI scores (5.8–3.0, *p* = 0.042) among adult patients. Qualitatively, the continuous data stream provides a safety net that reduces caregiver anxiety; Herbert et al. [7] noted that families described the technology as ‘less anxiety‐provoking’ than hospital‐based monitoring (which traditionally involves inpatient admissions for fasting challenges requiring frequent venous blood draws and hourly finger sticks), helping to ‘prevent burn‐out’ by validating the safety of home management. This integration of CGM to assess patient experience is increasingly formalized; in the gene therapy trial by Weinstein et al. [40], its utility was captured using Patient Global Impression of Change (PGIC) scores, marking a shift toward validated patient‐reported outcome measures.

Supporting these broader findings, anecdotal reports highlight CGM's psychological benefits in highly specific, high‐risk scenarios. Bonnet et al. [44] reported on a pregnant patient with GSD IIIa managed with CGM and telemonitoring (defined as weekly remote medical and dietetic consultations). The authors noted that CGM specifically reduced maternal fear and improved quality of life by providing reassuring alarms and continuous data that empowered the mother's self‐management. This allowed for targeted dietary adjustments and the safe discontinuation of nocturnal enteral feeding. Similarly, Ward et al. [36] noted that for the family of an infant with severe feeding aversion, CGM use ‘reduced the need for frequent finger stick checks’ and mitigated ‘severe family distress’. Broader evidence from inpatient studies quantifies this benefit, showing that CGM can reduce capillary fingerstick testing from approximately five checks per day to as few as two per day, thereby substantially decreasing the burden of painful procedures for patients and their caregivers [16].

CGM does not solve all sleep issues, especially in paediatric groups with high care burdens. Agnoletto et al. [32] found that despite optimal metabolic management, subjective sleep quality remained suboptimal in 80% of children studied, with actigraphy confirming shorter total sleep times and elevated daytime sleepiness. This suggests that while CGM lessens hypoglycemia concern, the physiological and behavioural demands of nocturnal GSD care, such as scheduled feeds or alarms, continue to affect sleep hygiene. Additionally, the physical burden of the device itself cannot be overlooked; Teufel‐Schäfer et al. documented a case of severe allergic contact dermatitis to several sensor adhesives that required discontinuation despite its glycemic benefits [47].

## 4 Discussion

### 4.1 The Paradigm Shift

CGM potentially can reframe hepatic GSD management from intermittent point‐of‐care checks to continuous risk surveillance, revealing clinically relevant hypoglycemia and variability that are frequently missed by SMBG. While Gugelmo et al. summarized CGM use across inherited metabolic disorders using a PubMed‐only approach [20], our multi‐database strategy enables a GSD‐focused synthesis of patterns that matter for diet titration, safety and trial endpoints. Beyond mere glucose observation, CGM actively drives the personalized adjustment of uncooked cornstarch (UCCS) doses and meal frequency. By utilizing sensor‐derived metrics, clinicians can iteratively refine dietary carbohydrate intake to minimize both nocturnal hypoglycemia and post‐prandial hyperglycemia, thereby preventing hepatic glycogen and lipid over‐storage [7, 12, 21].

Across cohorts and case‐based evidence, the consistent signal is asymptomatic instability, aligning with the recent stakeholder consensus that CGM's value is strongest when interpreted as trend data rather than isolated readings [12, 48]. Benchmarking against non‐diabetic reference profiles further supports a shift in goals toward reducing glycemic variability (CV often > 20% in GSD vs. ~17% in healthy participants) and increasing time in range, not simply avoiding overt symptomatic events [54, 55].

Despite these clear advantages, a major challenge in interpreting the current literature is the absence of a globally standardized consensus on optimal glucose targets specifically tailored for the GSD population. Unlike diabetes management, where unified guidelines exist [56, 57], current GSD CGM studies use heterogeneous euglycemia definitions. These include narrower healthy‐reference ranges, such as the stricter 70–140 mg/dL, versus broader diabetes‐derived time‐in‐range conventions such as 70–180 mg/dL (as the former may be considered a more physiological target that potentially yields better outcomes compared to the latter [58, 59, 60]). However, the available literature does not compare these thresholds head‐to‐head, so no evidence‐based preference for either range can yet be inferred. This heterogeneity complicates the direct comparison of clinical outcomes. Therefore, establishing uniform, GSD‐specific glycemic goals is essential for standardizing both future clinical trials and routine clinical practice.

### 4.2 Metabolic Impact and Safety Trade‐Offs

The clinical utility of CGM goes beyond glucose visualization to potentially improve secondary metabolic outcomes. Our review demonstrates that CGM‐guided dietary fine‐tuning directly correlates with reduced hepatic toxicity. Sensor‐based therapies lowered blood triglycerides and lactate in Kasapkara et al. and Sanli et al. [26, 38]. This shows that lowering glycemic excursions, specifically the counter‐regulatory surges triggered by subclinical hypoglycemia, stabilizes metabolic phenotype. Weinstein et al. recently validated CGM monitoring as a primary effectiveness objective in gene therapy trials, showing its sensitivity in capturing long‐term physiological homeostasis [40].

However, its acute safety device reliability must be differentiated from its chronic optimization utility. In the hypoglycemia range, CGM tends to overstate glucose levels, which may disguise actual lows [8]. Furthermore, marked hypertriglyceridemia can interfere with certain bedside capillary glucose methods, directly contributing to discordance between CGM and point‐of‐care (POC) testing. This discrepancy can be further exacerbated by critical illness and rapid fluctuations in blood glucose levels, which generally worsen the agreement between CGM readings and reference measurements [61, 62, 63]. Therefore, as a critical clinical recommendation, CGM cannot serve as a stand‐alone safeguard for acute events; confirmatory capillary testing remains mandatory when sensor readings fall below target thresholds (e.g., < 70 mg/dL) or when symptoms occur. In addition, algorithms developed around diabetic physiology are inherently tuned to insulin‐driven glucose dynamics. Consequently, they may not fully capture the fundamentally different pathophysiology of GSD, characterized by endogenous glucose production failure and much more abrupt hypoglycemic declines. This indicates that the limitation is not merely a matter of data scarcity, but a physiological divergence, reinforcing that CGM should be implemented within a hybrid safety framework [8, 21, 64].

### 4.3 The Human Element—Psychosocial Impact

Implementing CGM appear to alleviate caregiver anxiety and improves sleep, with significant PSQI improvements [22, 30]. Consistent with patient‐reported priorities, ‘peace of mind’ is the primary psychosocial benefit [12]. Despite CGM use, paediatric groups still have sleep disruption and daytime drowsiness [32] and alarm fatigue from false‐positive alerts remains a concern [64]. This alarm fatigue is often exacerbated by compression‐related sensor attenuations, where physical pressure on the device during sleep generates false‐positive hypoglycemic readings, further compromising sleep hygiene [65, 66, 67, 68]. Furthermore, the physical burden of the device itself must be acknowledged. Beyond rare cases of severe allergic contact dermatitis [47], broader evidence indicates that 20%–60% of paediatric and adult CGM users experience local pain, pruritus, or erythema at sensor sites [69, 70, 71, 72]. Ultimately, while CGM reduces the stress of physical invasiveness, it may shift the burden toward cognitive data monitoring. This necessitates targeted education and personalized alarm configurations to minimize unnecessary disruptions and prevent caregiver burnout.

### 4.4 Clinical Implementation and Future Directions

Implementation should be framed as subtype‐specific rather than purely glucocentric. This is because CGM glucose targets may look similar across subtypes, while non‐glucose morbidities, such as hyperlactatemia, hepatic adenomas and renal disease in GSD I, versus cardiomyopathy and progressive myopathy in GSD III, diverge significantly [52, 73]. Current global practice patterns already reflect this heterogeneity: a substantial proportion of centers recommend CGM broadly, while others restrict use to GSD I or to specific tasks such as dietary titration [74]. This approach is consistent with evidence that GSD III may retain abnormal transaminases and triglycerides despite comparable glycemic profiles, implying that CGM should be integrated with subtype‐relevant biomarkers to represent metabolic control more faithfully [23].

Despite this clinical utility, structural barriers persist. Derks et al. [12] identified reimbursement as the primary obstacle to standard‐of‐care implementation, though Herbert et al. [7] argue that sensor costs are likely offset by the reductions in emergency department visits and inpatient admissions for hypoglycemia. Looking forward, CGM is increasingly positioned as foundational infrastructure for next‐generation care, including its use as an efficacy endpoint in interventional trials [40]. Emerging machine‐learning methods trained on CGM signals support prediction and automation, but they must be prospectively validated in GSD‐specific physiology before becoming clinically reliable [75].

### 4.5 Strengths and Limitations

This review's strength is its methodological rigour and wide scope. We included six major databases (including Embase, Scopus and Cochrane), allowing for a more comprehensive evidence evaluation than Gugelmo et al. [20], which only searched PubMed. The limitations of the source literature restrict the strength of our conclusions. As noted in our risk of bias assessment, most included studies were observational, retrospective, single‐center case series with selection bias and no control groups. Evidence quality was inadequate due to small sample sizes (*n* < 20) typical of uncommon disease research, limiting generalizability. The variety of CGM devices (blinded professional vs. real‐time) and lack of uniform result reporting prevented a quantitative meta‐analysis. Additionally, a notable limitation in the current literature is the predominant reliance on older generations of CGM devices; there is a substantial lack of clinical studies evaluating the accuracy and efficacy of the latest technologies (e.g., Dexcom G7 or FreeStyle Libre 3) in the GSD population. Consequently, the findings presented in this systematic review should be considered primarily hypothesis‐generating rather than definitive evidence of efficacy. Furthermore, while CGM theoretically empowers patients by providing trend arrows and alarms for the early detection of impending severe hypoglycemia, there is a lack of robust quantitative data reporting the exact rates of severe hypoglycemic events before and after CGM initiation in the GSD population. Importantly, there are currently no large‐scale studies that systematically compare CGM readings with POC glucose alongside accurate blood lactate measurements. The absence of long‐term follow‐up data incorporating comprehensive metabolic markers limits the evidence base to fully support managing dietary transitions (e.g., extended‐release cornstarch) based solely on interstitial glucose values. To prove the long‐term clinical and economic value of continuous glucose monitoring in this vulnerable group, future research must prioritize multi‐center, prospective designs with standardized core outcome sets, and evaluate whether advanced sensor algorithms offer superior clinical benefits and better address the unique metabolic challenges associated with GSD management.

## 5 Conclusion

Current literature indicates that CGM has the potential to transform hepatic GSD management from reactive surveillance to proactive metabolic regulation, pending confirmation by larger, prospective trials. Current evidence shows that intermittent monitoring misses asymptomatic hypoglycemia, a major cause of long‐term hepatic and renal complications. CGM facilitates precise dietary and therapeutic titration, improving lipid profiles and lowering liver volume. Its utility is restricted by hypoglycemia sensor inaccuracy and false‐positive alerts' psychological impact. Thus, clinical implementation must prioritize trend analysis over isolated values and include non‐glucose indicators by subtype. Ultimately, validating CGM is the critical prerequisite for developing AI‐driven predictive models and automated delivery systems to reduce the disease burden.

## Author Contributions

**Reihaneh Mohsenipour:** data curation, investigation, formal analysis, writing – review and editing. **Asal Khalili Dehkordi:** writing – original draft, formal analysis, visualization, writing – review and editing. **Saeideh Abdolahpour:** supervision, methodology, writing – review and editing, validation. **Kasra Akbari:** conceptualization, methodology, investigation, project administration, writing – original draft, writing – review and editing. **Maryam Sadat Ghaderian:** investigation, resources, writing – review and editing, methodology. **Farzaneh Abbasi:** data curation, investigation, formal analysis, writing – review and editing.

## Funding

The authors have nothing to report.

## Ethics Statement

The authors have nothing to report.

## Consent

The authors have nothing to report.

## Conflicts of Interest

The authors declare no conflicts of interest.

## Supporting information


**Table S1:** Search Strings.

## Data Availability

The data that support the findings of this study are available within the article and its Table S1. Further inquiries can be directed to the corresponding author.
