# Supplementary material for: Continuous Glucose Monitoring in Glycogen Storage Diseases: A Systematic Review of Clinical Utility, Accuracy and Patient Outcomes
Source: Endocrinol Diabetes Metab. 2026 Jul 2;9(4):e70274. doi: 10.1002/edm2.70274 (PMC13329118; doi:10.1002/edm2.70274)
Supplement: Supplementary file 1 — Table S1: Search Strings. [file EDM2-9-e70274-s001.docx]

Table 1 - Search Strings

| **Database** | **Search String** |
| --- | --- |
| Pubmed (Medline) | ("Glycogen Storage Disease"[Mesh] OR "glycogen storage disease*"[Title/Abstract] OR GSD[Title/Abstract] OR "von Gierke"[Title/Abstract] OR "Pompe"[Title/Abstract] OR "Cori"[Title/Abstract] OR "Forbes"[Title/Abstract] OR "Andersen"[Title/Abstract] OR "McArdle"[Title/Abstract] OR "Hers"[Title/Abstract] OR "Tarui"[Title/Abstract] OR "Lewis"[Title/Abstract]) AND ("Continuous Glucose Monitoring"[Mesh] OR "Blood Glucose Self-Monitoring"[Mesh] OR CGM[Title/Abstract] OR "continuous glucose monitor*"[Title/Abstract] OR "flash glucose monitor*"[Title/Abstract] OR "intermittently scanned glucose monitor*"[Title/Abstract] OR FGM[Title/Abstract] OR "glucose sensor*"[Title/Abstract]) |
| Embase (Elsevier) | ('glycogen storage disease'/exp OR 'glycogen storage disease*':ti,ab,kw OR gsd*:ti,ab,kw OR 'von gierke':ti,ab,kw OR 'pompe':ti,ab,kw OR 'cori':ti,ab,kw OR 'forbes':ti,ab,kw OR 'andersen':ti,ab,kw OR 'mcardle':ti,ab,kw OR 'hers':ti,ab,kw OR 'tarui':ti,ab,kw OR 'lewis':ti,ab,kw) AND ('continuous glucose monitoring'/exp OR cgm:ti,ab,kw OR 'continuous glucose monitor*':ti,ab,kw OR 'flash glucose monitor*':ti,ab,kw OR 'intermittently scanned glucose monitor*':ti,ab,kw OR fgm:ti,ab,kw OR 'glucose sensor*':ti,ab,kw) |
| Scopus (Elsevier) | ( TITLE-ABS-KEY ( "glycogen storage disease*" ) OR TITLE-ABS-KEY ( GSD* ) OR TITLE-ABS-KEY ( "von Gierke" ) OR TITLE-ABS-KEY ( "Pompe" ) OR TITLE-ABS-KEY ( "Cori" ) OR TITLE-ABS-KEY ( "Forbes" ) OR TITLE-ABS-KEY ( "Andersen" ) OR TITLE-ABS-KEY ( "McArdle" ) OR TITLE-ABS-KEY ( "Hers" ) OR TITLE-ABS-KEY ( "Tarui" ) OR TITLE-ABS-KEY ( "Lewis" ) ) AND ( TITLE-ABS-KEY ( CGM ) OR TITLE-ABS-KEY ( "continuous glucose monitor*" ) OR TITLE-ABS-KEY ( "flash glucose monitor*" ) OR TITLE-ABS-KEY ( "intermittently scanned glucose monitor*" ) OR TITLE-ABS-KEY ( FGM ) OR TITLE-ABS-KEY ( "glucose sensor*" ) ) |
| Web of Science (Clarivate Analytics) | TS=("glycogen storage disease*" OR GSD* OR "von Gierke" OR "Pompe" OR "Cori" OR "Forbes" OR "Andersen" OR "McArdle" OR "Hers" OR "Tarui" OR "Lewis") AND TS=(CGM OR "continuous glucose monitor*" OR "flash glucose monitor*" OR "intermittently scanned glucose monitor*" OR FGM OR "glucose sensor*") |
| Cochrane Library (Wiley) | #1 ("glycogen storage disease") OR (glycogen NEXT storage NEXT disease*) OR GSD* OR "von Gierke" OR "Pompe" OR "Cori" OR "Forbes" OR "Andersen" OR "McArdle" OR "Hers" OR "Tarui" OR "Lewis"  #2 CGM OR (continuous NEXT glucose NEXT monitor*) OR (flash NEXT glucose NEXT monitor*) OR (intermittently NEXT scanned NEXT glucose NEXT monitor*) OR FGM OR (glucose NEXT sensor*)  #3 #1 AND #2 |
